# Effects of homologous oviductal epithelial cell co-culture on in vitro embryo development in livestock species: A systematic review and meta-analysis protocol

**DOI:** 10.1371/journal.pone.0360151

**Published:** 2026-10-06

**Authors:** Muloongo C. Sitali, Mawiini Mwandabantu, Madalitso Chelenga

**Affiliations:** 1 Department of Biomedical Sciences, School of Veterinary Medicine, The University of Zambia, Lusaka, Zambia; 2 Department of Clinical Studies, School of Veterinary Medicine, The University of Zambia, Lusaka, Zambia; 3 Department of Veterinary Clinical Studies, Faculty of Veterinary Medicine, The Lilongwe University of Agriculture and Natural Resources, Lilongwe, Malawi; School of Sciences and Languages, Sao Paulo State University (UNESP), BRAZIL

## Abstract

**Purpose:**

The objective of this protocol is to outline the approach for a systematic review and meta-analysis aimed at evaluating the effects of homologous oviductal epithelial cell (OEC) co-culture on *in vitro* embryo developmental outcomes, including cleavage, blastocyst, expanded blastocyst, and hatched blastocyst development, as well as blastocyst quality assessed by total blastocyst cell number, in livestock species.

**Methods:**

The protocol is developed following the Preferred Reporting Items for Systematic Reviews and Meta-Analyses Protocols (PRISMA-P). The protocol has been registered in PROSPERO (CRD420261405630). The approach is based on the Population, Intervention, Comparator, Outcome and Study design (PICOS) framework to answer the following research questions: 1) Does homologous oviductal epithelial cell (OEC) co-culture with oocytes, presumptive zygotes or embryos improve *in vitro* embryo developmental outcomes compared with culture systems without OEC co-culture in sheep, cattle, pigs, and goats? 2) What are the factors influencing the effectiveness of the OEC co-culture systems, such as the stage of OEC inclusion in the embryo production system, i.e., during oocyte maturation, fertilization, embryo culture, or combined stages? The process will include: 1) development of the search strategy and inclusion criteria; 2) data extraction; 3) risk of bias assessment; and 4) data analysis. The risk of bias of the included *in vitro* studies will be evaluated using the National Toxicology Program-Office of Health Assessment and Translation (NTP-OHAT) Risk of Bias Tool. Given the anticipated heterogeneity in study designs, species, OEC models, and culture conditions, meta-analyses will be conducted using a random-effects model, with pooled effect estimates calculated using the inverse-variance method. Species will be assessed as a main effect, whereas subgroup analyses will be performed according to the oviductal cell co-culture systems and/or the stage at which OEC co-culture is introduced within the *in vitro* embryo production system. For studies where numerical data are insufficient for meta-analysis or cannot be extracted, findings will be synthesized descriptively. Publication bias will be assessed using funnel plots and, where appropriate, Egger’s regression test, while sensitivity analyses will be conducted using the leave-one-out method.

**Conclusions:**

This systematic review and meta-analysis will provide a comprehensive synthesis of the evidence on the effect of OEC-oocyte/embryo co-culture systems. The findings will help clarify the current state of knowledge, identify key research gaps, provide a foundation for future studies aimed at optimizing *in vitro* embryo production systems, and improve our understanding of potential sources of heterogeneity across studies.

## Introduction

Embryo production under *in vitro* conditions constitutes a key aspect of modern reproductive biotechnology [1] and is integral to livestock breeding, genetic improvement and conservation initiatives [2,3]. Despite significant progress in *in vitro* embryo production (IVP) systems, *in vitro* produced embryos frequently exhibit reduced developmental competence and compromised quality relative to embryos produced *in vivo* [1,4]. Although IVP systems can support oocyte maturation, fertilization, and embryo development to the blastocyst stage, suboptimal culture conditions throughout these stages may impair developmental competence, embryo quality, viability [5,6], and subsequent implantation potential [4,7]. Co-culture of oocytes [8,9] and embryos [10,11] with oviductal epithelial cells (OECs) during *in vitro* maturation (IVM), *in vitro* fertilization (IVF) and *in vitro* culture (IVC), respectively, has been explored as an alternative to conventional culture systems to improve the developmental competence of oocytes *in vitro* [12].

The biological significance of the oviduct during early embryonic development is demonstrated by studies showing that zygotes cultured within recipient cattle oviducts [13], sheep oviducts [14] or isolated mouse oviducts [15] yield embryos of higher quality than those cultured in conventional *in vitro c*ulture systems*.* Contemporary research further indicates that simulating the oviductal environment through OEC co-culture or by incorporating physiological oviductal fluids, can enhance bovine embryo quality and developmental competence [16–18]. Conventional IVP systems, while capable of supporting fertilization and embryo development, are generally performed without OECs and may consequently lack many cellular and molecular signals present in the natural oviductal microenvironment. Additionally, OECs have been reported to modulate sperm function and maintain sperm viability within the oviductal environment [19], potentially contributing to improved fertilization outcomes and subsequent embryo development [20]. Collectively, these findings indicate that the oviduct provides critical biochemical and cellular cues not fully replicated by conventional culture systems [12].

The oviduct provides the first maternal environment encountered by the embryo after fertilization and plays a critical role in regulating early embryonic development [21,22]. OECs contribute to this biological milieu through direct cell-to-cell interactions and the secretion of numerous bioactive molecules, including glycoproteins and complex carbohydrates [23], that play a supportive role in embryo survival, development, and quality [24]. Consequently, OEC-based culture systems have been incorporated into IVP protocols to better mimic physiological oviductal conditions and enhance developmental outcomes. Various approaches, including monolayers, spheroids, aggregates, and explants, have been investigated [10,25,26]. However, findings remain inconsistent, likely due to differences in species, culture conditions, OEC source, oviduct region, OEC inclusion stage, and other methodological factors.

Although OEC-based culture systems have been explored in experimental embryo production, their overall effectiveness varies and remains inconclusive across livestock species. Furthermore, it remains unclear whether factors such as species and the stage of OEC inclusion in co-culture systems contribute to variation in embryonic developmental outcomes. To our knowledge, no comprehensive evidence synthesis evaluating the effects of homologous OEC co-culture systems has been conducted to date. Therefore, the prospective systematic review and meta-analysis aim to evaluate the effects of homologous OEC co-culture with oocytes, presumptive zygotes or embryos during IVM, IVF, IVC, or combined stages on *in vitro* developmental competence and blastocyst quality (assessed by total cell number) in bovine, ovine, caprine, and porcine species. Additionally, the influence of the oviductal cell co-culture system and stage of OEC inclusion within the IVP system on these developmental outcomes will be evaluated. By quantitatively synthesizing the available evidence and exploring potential sources of heterogeneity across studies, this review will identify conditions under which OEC co-culture may be most beneficial, highlight existing knowledge gaps, and provide evidence-based recommendations to guide future research and enhance IVP technologies in livestock species.

## Methods

This systematic review and meta-analysis was prospectively registered in PROSPERO (CRD420261405630**).** Any amendments to the protocol will be acknowledged in the PROSPERO registration and in the subsequent systematic review publication. The review protocol was developed in accordance with the Preferred Reporting Items for Systematic Review and Meta-Analysis Protocols (PRISMA-P) guidelines [27]. The review process includes: (1) development of the search strategy; (2) application of predefined inclusion and exclusion criteria; (3) data extraction; (4) assessment of risk of bias; and (5) data synthesis and analysis. Literature searching, study selection, data extraction, and statistical analyses will be conducted in accordance with the recommendations outlined in the Cochrane Handbook for Systematic Reviews of Interventions (Version 6.5) [28]. Meta-analyses will be performed using the Meta-Analysis procedure in the Statistical Package for the Social Sciences (SPSS) Version 31 (IBM Corp., Armonk, NY, USA).

### Search strategy

A comprehensive literature search strategy was developed to identify controlled experimental studies evaluating the effects of homologous oviductal epithelial cell (OEC) co-culture on mammalian embryo development under *in vitro* culture conditions. Searches will be conducted in PubMed, ScienceDirect, CAB Abstracts, Scopus, and the Wiley Online Library. The search string ((“oviductal epithelial cells” OR “oviduct epithelium” OR “tubal epithelial cells” OR “ampullary epithelial cells” OR “ampulla epithelial cells” OR “isthmic epithelial cells” OR “isthmus epithelial cells” OR “whole oviduct” OR OEC OR BOEC OR POEC OR GOEC OR COEC OR OOEC) AND (embryo OR blastocyst OR “cleaved embryos” OR “embryonic development” OR “embryo culture”) AND (bovine OR cattle OR cow OR ovine OR sheep OR ewe OR porcine OR pig OR sow OR swine OR caprine OR goat)), will be used. The search strategy will be adapted as necessary to accommodate the search functionalities and indexing systems of individual databases. In addition to the electronic database searches, the reference lists of all included studies and relevant review articles will be manually screened to identify additional potentially eligible studies not captured by the database searches.

### Inclusion and exclusion criteria

The inclusion and exclusion criteria will be defined according to the Population, Intervention, Comparator, Outcomes, and Study Design (PICOS) framework (Table 1). Only peer-reviewed original research articles published in English, or articles that can be adequately translated into English, will be included. Studies for which a reliable translation cannot be obtained will be excluded to minimize the risk of data extraction errors and misclassification bias. Studies published between January 1980 and April 2026 will be considered for inclusion. Reviews, editorials, commentaries, letters, case reports, and conference abstracts will be excluded. Studies published before January 1980 or after April 2026, those lacking a clearly defined non-OEC control group, studies involving species other than bovine, ovine, caprine, and porcine, and those from which relevant outcome data cannot be extracted or calculated will also be excluded. Additionally, to minimize the risk of inaccurate estimation, studies reporting outcome data solely in graphical form, from which numerical values cannot be reliably extracted or calculated, will be excluded from the quantitative analysis.

**Table 1:** Eligibility criteria based on the PICOS framework.

Component	Description
**Population (P)**	Mammalian oocytes, presumptive zygotes and embryos from bovine, ovine, caprine, and porcine species cultured *in vitro*
**Intervention (I)**	Homologous OEC co-culture systems (monolayers, spheroids, aggregates, clusters, explants)
**Comparator (C)**	Culture systems without OEC co-culture
**Outcomes (O)**	Cleavage rate, blastocyst rate, expanded blastocyst rate, hatched blastocyst rate, and total blastocyst cell number
**Study Design (S)**	Controlled experimental studies evaluating OEC co-culture under *in vitro* conditions

Two reviewers (Muloongo Chinani Sitali and Mawiini Mwandabantu) will use Covidence to perform study screening and selection. After duplicate records are removed, the two reviewers will independently screen titles and abstracts. Thereafter, potentially eligible full-text articles will be retrieved and independently assessed against the predefined inclusion and exclusion criteria. Disagreements will be resolved through discussion or, when necessary, consultation with a third reviewer.

### Data extraction

For each eligible study, data will be extracted on the first author, year of publication, study objective, embryo production method, culture conditions, sample size, stage of OEC inclusion (during oocyte maturation, fertilization, or embryo culture), species, type of semen used, oviduct region used (ampulla, isthmus, or whole oviduct), estrous cycle stage of the oviduct (e.g., follicular, luteal, or estrus), OEC model (e.g., monolayer, spheroid, or aggregates), culture medium, and any additional supplements or modifications applied during embryo production and the day of blastocyst rate assessment (i.e., Day 7, Day 8, or Day 9). Outcome data will include event counts and/or percentages for cleavage, blastocyst (early, expanded and hatched stages), and total number of cells in blastocysts. Where raw event counts are unavailable, they will be calculated from the reported percentages and corresponding sample sizes for each group, provided that the denominator is clearly defined. For the total blastocyst cell number, mean, standard deviation (SD), standard error (SE), and the number of blastocysts assessed will be extracted. For studies containing multiple OEC treatment groups compared with a shared control group, the control group sample size will be divided equally among the relevant comparisons to avoid double-counting experimental units and overestimating the precision of the pooled effect estimate, in accordance with Cochrane recommendations for pairwise meta-analysis [28].

### Risk of bias assessment

The risk of bias of the included studies will be assessed using criteria adapted from the National Toxicology Program-Office of Health Assessment and Translation (NTP-OHAT) risk of bias tool for the evaluation of *in vitro* studies [29]. Two independent reviewers will assess each study, with disagreements resolved through discussion or consultation with a third reviewer. The assessment will consider the following risk of bias domains: (1) randomization of experimental units; (2) comparability of experimental conditions; (3) completeness of outcome data; (4) exposure characterization; (5) outcome assessment; (6) selective reporting; and (7) other bias. For each domain, studies will be classified as definitely low risk, probably low risk, definitely high risk and probably high risk of bias.

### Quality assessment of the included studies

The methodological quality and completeness of reporting of the included studies will be assessed using a framework based on the Critical Appraisal of Methodological Quality, Quality of Reporting, and Risk of Bias in Animal Research (CRIME-Q) guidelines [30]. The framework domains will address methodological features specific to bench-top and laboratory OEC-embryo co-culture studies. The assessment will consider: (1) OEC source and characterization; (2) adequate description of OEC culture conditions; (3) description of the co-culture procedure; (4) appropriateness of the technical approach; (5) appropriate control group; (6) description of gamete or embryo source; (7) experimental conditions adequately described; (8) sample size and replication; (9) allocation and baseline comparability; (10) definition and assessment of outcomes and time points; (11) appropriateness of statistical methods and reporting of funding and conflicts of interest. Each domain will be rated as yes, no, partly, or unclear.

### Data analysis

Outcomes will be categorized as primary outcomes (cleavage rate and blastocyst rate) and secondary outcomes (expanded blastocyst rate, hatched blastocyst rate, and total blastocyst cell number). Cleavage rate will be defined as the proportion of inseminated oocytes that undergo cleavage, while blastocyst rate will be defined according to the denominator used in the primary study. Blastocyst rate per inseminated oocyte and blastocyst rate per cleaved embryo will be treated as separate outcomes and will not be pooled. Expanded and hatched blastocyst rates will similarly be analyzed according to the denominator reported in the primary studies, while total blastocyst cell number will be expressed as the mean number of cells per blastocyst. Where at least two sufficiently comparable studies are available [31,32], meta-analyses will be performed to quantitatively synthesize the evidence from eligible controlled experimental studies. For the total blastocyst cell number, pooled effects will be estimated using either the mean difference (MD) or standardized mean difference (SMD) with 95% confidence intervals, as appropriate. Risk ratios (RRs) with 95% confidence intervals (CIs) will be calculated for dichotomous outcomes and pooled using the inverse-variance method under a random-effects model to account for expected methodological and biological heterogeneity among studies [28].

Statistical heterogeneity will be assessed using Cochran’s Q test and quantified using the inconsistency (I²) metric. Heterogeneity will be interpreted as follows: I^2^ < 30% (low heterogeneity), I^2^ of 30–50% (moderate heterogeneity), and I^2^ > 50% (substantial heterogeneity) [33,34]. Species will be evaluated as a main effect on embryo developmental outcomes, while subgroup analyses will be conducted according to the stage of OEC inclusion within the embryo production system and/or oviductal cell co-culture system where at least two studies are available [31]. Publication bias will be assessed through visual inspection of funnel plot asymmetry [34,35], while Egger’s regression test will be performed only when at least 10 studies are available for a given outcome [36]. Statistical significance will be considered at *p* < 0.05. Sensitivity analyses will be performed to evaluate the stability and robustness of the pooled outcomes by sequentially excluding individual studies (leave-one-out analysis) and applying iterative outlier detection methods. Meta-analyses will be performed using the Meta-Analysis procedure in IBM SPSS Statistics Version 31 (IBM Corp., Armonk, NY, USA).

### The status and timeline of the study

The review is planned, with completion and dissemination of the findings anticipated within five months.

## Discussion

This systematic review and meta-analysis is expected to provide a comprehensive quantitative assessment of the effects of homologous oviductal epithelial cell (OEC) co-culture systems on embryo development in bovine, ovine, caprine, and porcine species. By synthesizing data from individual studies, the review will provide a more robust evaluation of the impact of OEC co-culture on cleavage, blastocyst, expanded blastocyst, and hatched blastocyst development, as well as blastocyst quality assessed by total blastocyst cell number than can be achieved from individual experiments alone. To ensure the generation of high-quality evidence, the review protocol has been developed in accordance with established guidelines for systematic reviews and meta-analyses. A comprehensive search strategy has been designed to identify relevant studies from electronic databases, while predefined eligibility criteria, duplicate independent screening, standardized data extraction procedures, and structured risk-of-bias assessment will be used to minimize bias throughout the review process. In addition, quantitative synthesis and predefined subgroup analyses will facilitate the exploration of variation in treatment effects and improve understanding of factors that may influence the response to OEC co-culture.

This protocol has several strengths that enhance the biological relevance and methodological rigor of the proposed review. Limiting eligibility to homologous co-culture systems, in which OECs and oocytes or embryos are derived from the same species, reduces variability associated with heterologous models and enhances the biological relevance of comparisons. Furthermore, the protocol specifies evaluation of the developmental stage at which OECs are introduced during IVM, IVF, IVC, or combined stages, allowing assessment of whether the effectiveness of OEC co-culture varies according to the stage of exposure. The protocol also includes comprehensive extraction of methodological variables, such as OEC model, oviduct region, estrous-cycle stage, culture conditions, semen type, and embryo production procedures, which facilitates interpretation of potential sources of heterogeneity. Additionally, assessment of multiple developmental endpoints, including cleavage, blastocyst development, blastocyst expansion and hatching, together with total blastocyst cell number, provides a broader evaluation of the effects of OEC co-culture across different stages of embryo development than reliance on a single developmental endpoint. Together, these approaches are expected to facilitate a more precise assessment of the influence of OEC co-culture on embryo development. Nevertheless, this review is subject to several limitations which should be considered when interpreting the findings. Differences in embryo production protocols, culture conditions, estrus cycle stage of the oviducts, and other methodological characteristics may contribute to substantial heterogeneity and reduce comparability among studies. Furthermore, incomplete reporting of experimental procedures may affect both the assessment of risk of bias and the interpretation of pooled effect estimates. Besides, the detection of publication bias may be limited in some subgroup analyses because funnel plots and Egger's regression test have limited statistical power when fewer than ten studies contribute to an analysis, which may reduce the ability to reliably detect small-study effects or publication bias. These factors will be carefully considered when concluding the available evidence.

As research on OEC-based culture systems continues to expand, this review will help to identify knowledge gaps and highlight areas requiring further investigation. The findings may have important implications for reproductive biotechnology research and practice by identifying effective applications of OEC co-culture and informing the development of more physiologically relevant embryo culture strategies. In addition, the review may support efforts to improve *in vitro* embryo production systems and guide future studies aimed at enhancing embryo developmental competence and quality in livestock species.

## Conclusion

This systematic review will synthesize the available evidence and identify knowledge gaps regarding the use of homologous OEC co-culture systems in embryo production in livestock species. Findings from the meta-analysis will provide guidance for optimizing OEC-based culture strategies and designing future studies aimed at improving embryo developmental outcomes under *in vitro* conditions**.**

## Supporting information

S1 Checklist PRISMA 2020 checklist. (DOCX)
